# Resection of Olfactory Groove Meningiomas Through Unilateral vs. Bilateral Approaches: A Systematic Review and Meta-Analysis

**DOI:** 10.3389/fonc.2020.560706

**Published:** 2020-10-22

**Authors:** Austin Y. Feng, Sandy Wong, Sabir Saluja, Michael C. Jin, Anthony Thai, Arjun V. Pendharkar, Allen L. Ho, Prasad Reddy, Allen D. Efron

**Affiliations:** ^1^Department of Neurosurgery, Stanford University School of Medicine, Stanford, CA, United States; ^2^Department of Neurosurgery, Kaiser Permanente, Redwood City, CA, United States

**Keywords:** olfactory groove meningioma, transcranial approach, complications, meta- analysis, systematic review

## Abstract

**Introduction:** Consensus is limited regarding optimal transcranial approaches (TCAs) for the surgical resection of olfactory groove meningiomas (OGMs). This systematic review and meta-analysis aims to examine operative and peri-operative outcomes of unilateral compared to bilateral TCAs for OGMs.

**Methods:** Electronic databases were searched from inception until December 2019 for studies delineating TCAs for OGM patients. Patient demographics, pre-operative symptoms, surgical outcomes, and complications were evaluated and analyzed with a meta-analysis of proportions.

**Results:** A total of 27 observational case series comparing 554 unilateral vs. 451 bilateral TCA patients were eligible for review. The weighted pooled incidence of gross total resection is 94.6% (95% CI, 90.7–97.5%; *I*^2^ = 59.0%; *p* = 0.001) for unilateral and 90.9% (95% CI, 85.6–95.4%; *I*^2^ = 58.1%; *p* = 0.003) for bilateral cohorts. Similarly, the incidence of OGM recurrence is 2.6% (95% CI, 0.4–6.0%; *I*^2^ = 53.1%; *p* = 0.012) and 4.7% (95% CI, 1.4–9.2%; *I*^2^ = 55.3%; *p* = 0.006), respectively. Differences in oncologic outcomes were not found to be statistically significant (*p* = 0.21 and 0.35, respectively). Statistically significant differences in complication rates in bilateral vs. unilateral TCA cohorts include meningitis (1.0 vs. 0.0%; *p* = 0.022) and mortality (3.2 vs. 0.2%; *p* = 0.007).

**Conclusions:** While both cohorts have similar oncologic outcomes, bilateral TCA patients exhibit higher post-operative complication rates. This may be explained by underlying tumor characteristics necessitating more radical resection but may also indicate increased morbidity with bilateral approaches. However, evidence from more controlled, comparative studies is warranted to further support these findings.

## Introduction

Olfactory groove meningiomas (OGMs) are arachnoid cell neoplasms of the frontoethmoidal suture and lamina cribrosa, accounting for 4.5–18% of intracranial meningiomas (1). Arising along the midline of the anterior fossa, OGMs frequently impinge on the frontal lobes through mass effect. Presenting symptoms vary but commonly begin with ipsilateral anosmia that is difficult to detect. As the growth enlarges, displacement of adjacent brain regions leads to headache, fatigue, seizures, and intracranial hypertension. Of note is that the compression of the optic chiasm may lead to visual acuity defects. Nevertheless, due to frontal lobe plasticity and their insidious growth, OGMs can grow substantially prior to symptom onset. Though histologically classified as benign tumors, OGMs can still have a detrimental effect on a patient's well-being and quality of life.

Traditionally, surgical resection of OGMs has been achieved through transcranial approaches (TCAs). These encompass a plethora of routes including subfrontal, subcranial, interhemispheric, pterional, etc. (2–5). While newer techniques, such as endoscopic endonasal approach (EEA), have been introduced, TCAs remain a core component of the armamentarium for large OGM management due to their variety, size, and difficulty of extensive endoscopic repair of the anterior skull base (6). However, few studies have compared outcomes and complications between different TCAs. In 2007, Nakamura et al. investigated the differences in outcome following bifrontal, unilateral, and pterional approaches on 82 patients (7). In the largest case series of its kind, Pallini et al. compared bifrontal, fronto-orbito-basal, and pterional approaches among 99 patients in 2015 (8). Though these were important observational studies, their insights are limited in scope as single-institution case series.

While multiple meta-analyses have compared EEA and TCA, none have been performed for specific TCAs. To our knowledge, this is the first study to perform a systematic review and meta-analysis of the literature examining unilateral vs. bilateral approaches for OGM resection. Understandably, there are certain analytical obstacles. Most studies investigating OGMs are case reports, and there are no randomized controlled trials (RCTs); direct comparative studies of TCAs are also scarce. The diversity of TCAs also introduces additional complexity. To bypass these issues, we propose categorizing TCAs into either bilateral or unilateral approaches to simplify moderator analysis and to have a sufficient number of studies per category. While this method can limit the analyses on each specific TCA, the meta-analytical insights regarding approach laterality may contribute a broader perspective to help guide debate on optimal OGM treatment.

## Methods

### Search Strategy

This systematic review was conducted using Preferred Reporting Items for Systemic Reviews and Meta-Analyses (PRISMA) guidelines and recommendations. Searches were performed on PubMed, SCOPUS, Embase, Web of Science, and Medline databases on all publications before December 2019. The literature was reviewed with the following MeSH terms in all permutations: “meningioma” AND “olfactory” AND “groove.” The reference lists of articles were further examined to identify potentially relevant articles. All retrieved studies were independently reviewed by two investigators (AF and SS) and assessed according to inclusion and exclusion criteria.

### Selection Criteria

Studies eligible for inclusion had patients undergoing OGM resection and reported post-operative complications and outcomes. Case studies, series with fewer than 10 OGM patients, indiscernible cohorts of surgical approaches and/or mixed pathologies, and studies with unclear outcomes or complications were excluded. Only English-language publications were screened. Abstracts, technical reports, cadaver studies, conference presentations, reviews, and editorials were also excluded.

### Data Extraction and Appraisal

All data were extracted from the articles' tables, figures, and texts. Any estimate measures were based on original data and used validated statistical methodology (9–11). The investigators (AF and SS) independently reviewed and performed extraction on each retrieved article; discrepancies were resolved through discussion and consensus. The data extracted include patient demographics (e.g., sex and age), pre-operative symptoms (e.g., anosmia, vision defects, headache, seizure, etc.), surgical approach, tumor volume, resection outcome, post-operative visual outcome, complications [e.g., hydrocephalus, infection, cerebrospinal fluid (CSF) leak, etc.], mean follow-up, and recurrence rate. Study quality was appraised by two investigators (AF and SW) according to a critical review checklist of the Dutch Cochrane Center proposed by the Meta-analysis of Observational Studies in Epidemiology group.

### Statistical Analysis

Meta-analysis of proportions was performed for pre-operative symptoms and post-operative complications. To stabilize the variance of observed proportions, a double-arcsine (Freeman–Tukey) transformation was applied. Random effects (RE) models estimated by the DerSimonian–Laird method were used to combine transformed proportions to incorporate heterogeneity. Pooled estimates were back-transformed. Heterogeneity was tested and quantified by Cochran *Q* and *I*^2^ tests, respectively. Study effect sizes are weighted by the inverse of their variance. Analyses were performed using the metafor and meta packages for R version 3.6.3. Statistical significance is established at *p*-value <0.05. Assessment of potential publication bias is achieved through funnel plots, Begg rank correlation test, and Egger's test.

## Results

### Literature Search Results

**Figure 1:**
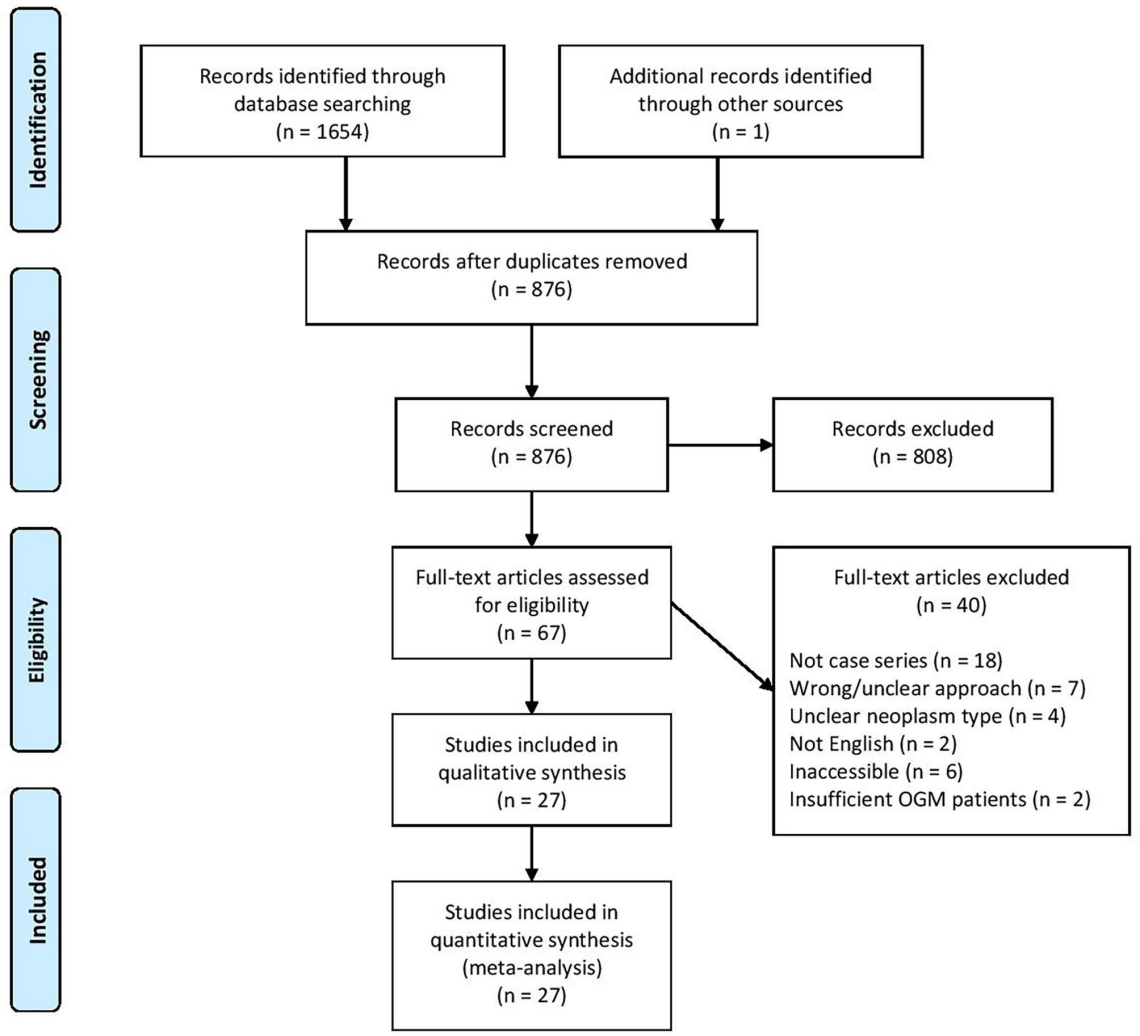
Study selection according to Preferred Reporting Items for Systematic Reviews and Meta-Analyses guidelines.

The search terminology yielded a total of 1,655 articles from various electronic databases and additional sources like reference lists. After duplicates were removed, 876 articles remained. Application of inclusion and exclusion criteria ultimately identified 27 studies for further data extraction and meta-analysis (Table 1). These studies span from 2019 to 1996 and come from 13 countries. All studies were retrospective case series. A total of 24 studies exclusively reported on OGM, while three studies also included other neoplasms. The literature search process is diagrammed in Figure 1.

**Table 1:** Study characteristics.

**References**	**Year**	**Transcranial approach category**	**Country**	**Design**	**Study period**	**Specific transcranial approach**
Patel et al. (12)	2019	B	UK	Retrospective case series	2002–2016	Bifrontal transbasal, bifrontal interhemispheric
Xu et al. (13)	2019	B	China	Retrospective case series	2013–2017	Small extended bifrontal
Farooq et al. (14)	2018	B	Pakistan	Retrospective case series	N/A	Bicoronal, subfrontal without orbital osteotomies
Liu et al. (15)	2018	B	USA	Retrospective case series	2007–2016	Transbasal
Guduk et al. (2)	2017	U	Turkey	Retrospective case series	1987–2015	Pterional, unifrontal
Barzaghi et al. (16)	2017	B	Italy	Retrospective case series	2001–2014	Transfrontal-sinus-subcranial
Nanda et al. (17)	2016	B/U	USA	Retrospective case series	1990–2014	Bifrontal, fronto-orbito-basal, frontolateral, pterional
de Alemeida et al. (18)	2015	B	USA/Canada	Retrospective case series	2003–2012	Bifrontal
Pallini et al. (8)	2015	B/U	Italy	Retrospective case series	1984–2010	Bifrontal, fronto-orbito-basal, pterional
Mielke et al. (3)	2014	U	Germany	Retrospective case series	1990–2013	Anterior interhemispheric
Refaat et al. (19)	2014	B/U	Egypt	Retrospective case series	2012–2013	Bifrontal basal interhemispheric, bilateral subfrontal, frontotemporal, unilateral subfrontal
Bitter et al. (20)	2013	U	Germany	Retrospective case series	1991–2010	Pterional
Jang et al. (21)	2013	B/U	Korea	Retrospective case series	1993–2012	Bifrontal, frontolateral
Musluman et al. (22)	2012	U	Turkey	Retrospective case series	1996–2008	Unilateral subfrontal interhemispheric transfalcial
Tomasello et al. (23)	2011	U	Italy	Retrospective case series	1991–2007	Pterional
Pepper et al. (24)	2011	B	USA	Retrospective case series	1995–2009	Transglabellar/subcranial
El-Bahy et al. (25)	2009	U	Egypt	Retrospective case series	2003–2008	Frontolateral
Aguiar et al. (26)	2009	B/U	Brazil	Retrospective case series	1997–2007	Bifrontal, fronto-orbital, fronto-pterional
Romani et al. (4)	2009	U	Finland	Retrospective case series	1997–2008	Lateral supraorbital
Gazzeri et al. (27)	2008	B	Italy	Retrospective case series	1990–2004	Bifrontal
Colli et al. (28)	2007	B	Brazil	Retrospective case series	1988–2006	Bifrontal, bifrontal-bi-orbital
Nakamura et al. (7)	2007	B/U	Germany	Retrospective case series	1972–2002	Bifrontal, pterional, unilateral subfrontal
Spektor et al. (5)	2005	B/U	Israel	Retrospective case series	1990–2003	Bifrontal, fronto-orbital, pterional, subcranial, unilateral subfrontal
Paterniti et al. (29)	1999	U	Italy	Retrospective case series	1975–1996	Pterional
Turazzi et al. (30)	1999	U	Italy	Retrospective case series	1989–1996	Pterional
Tsikoudas et al. (31)	1999	B	UK	Retrospective case series	1976–1998	Bifrontal
Mayfrank et al. (32)	1996	U	Germany	Retrospective case series	N/A	Frontal interhemispheric

*U, unilateral; B, bilateral*.

### Demographics

Selected studies encompassed 1,005 subjects overall, with 554 and 451 receiving unilateral and bilateral approaches, respectively. Females are 65.3% of the subject population, with 64.8 and 66.1% receiving unilateral and bilateral approaches. The average age, weighted by study sample size, is 57.4 years in the unilateral cohort and 56.2 years in the bilateral cohort. Under unilateral, specific approaches include pterional/frontotemporal, unilateral subfrontal, interhemispheric, and lateral supraorbital. For bilateral, specific approaches include bifrontal, bifrontal variations (transbasal, interhemispheric, extended), subfrontal, subcranial, and fronto-orbito-basal. The weighted mean follow-up period for the unilateral cohort is 69.9 months and for the bilateral cohort is 74.0 months (Table 2).

**Table 2:** Demographics and pre-operative symptoms.

**References**	**Transcranial approach category**	**Size (*n*)**	**Demographics**		**Mean follow-up (months)**	**Tumor volume (cm^**3**^)**	**Pre-operative symptoms**					
			**Age**	**Females**			**Anosmia**	**Visual issues**	**Headache**	**Seizure**	**Behavioral abnormality**	**Fatigue**
Patel et al. (12)	B	48	62	36	59	49	18	18	17	11	24	0
Xu et al. (13)	B	29	55	18	40	43	17	6	0	4	15	0
Farooq et al. (14)	B	19	51	18	60	113	11	15	15	0	13	0
Liu et al. (15)	B	15	52	10	14.5	92	0	4	3	2	2	0
Guduk et al. (2)	U	61	58	43	N/A	62	9	14	16	6	9	0
Barzaghi et al. (16)	B	21	54	12	87	51	11	8	0	3	8	0
Nanda et al. (17)	B/U	16/41	55/60	7/27	59.6	N/A	9/21	6/16	8/25	2/5	8/17	6/14
de Alemeida et al. (18)	B	10	50	8	N/A	36	N/A	N/A	N/A	N/A	N/A	N/A
Pallini et al. (8)^*^	B/U	81/18	57	52/12	103	67/70	59	46	38	19	35	0
Mielke et al. (3)	U	43	62	27	N/A	N/A	27	14	0	0	22	0
Refaat et al. (19)	B/U	8/6	54/47	6/5	N/A	134/69	4/3	4/3	6/5	0/1	2/2	0/0
Bitter et al. (20)	U	61	60	40	122	N/A	30	22	18	5	16	3
Jang et al. (21)	B/U	19/21	55/53	7/10	58	60.1/41.6	17/12	0/0	0/0	0/0	0/0	0/0
Musluman et al. (22)	U	42	59	24	52	N/A	19	23	37	10	28	0
Tomasello et al. (23)	U	18	59	12	93.5	23	18	13	13	2	18	0
Pepper et al. (24)	B	19	51	8	N/A	96	1	5	8	1	6	2
El-Bahy et al. (25)	U	18	49	10	31	34	13	5	18	3	8	0
Aguiar et al. (26)	B/U	7/14	55.5	15	50	41.6	21	8	12	5	4	0
Romani et al. (4)	U	66	57	38	45	54	38	22	11	14	33	0
Gazzeri et al. (27)	B	36	56	24	111	137	30	20	18	10	25	0
Colli et al. (28)	B	17	53	16	51	N/A	5	0	11	5	0	0
Nakamura et al. (7)^*^	B/U	46/36	58	63	63.4	61/38	48	20	26	16	59	0
Spektor et al. (5)^*^	B/U	47/34	55	58	89/71	48/47	47	22	41	9	21	0
Paterniti et al. (29)	U	20	49	15	N/A	N/A	N/A	N/A	N/A	N/A	N/A	N/A
Turazzi et al. (30)	U	37	N/A	N/A	48	99	27	16	0	0	27	0
Tsikoudas et al. (31)	B	13	60	10	N/A	113	4	5	8	2	8	1
Mayfrank et al. (32)	U	18	N/A	13	N/A	18	11	4	0	1	10	0

* *Not used in pre-operative symptom calculations because the values were not differentiated between approach categories*.

### Pre-operative Symptoms

The most common pre-operative symptoms in the unilateral and the bilateral cohorts are anosmia (54.1/48.5%) and behavioral anomalies (43.2/42.4%), respectively. The least common pre-operative symptoms are fatigue (3.8/3.4%) and seizures (11.2/15.3%) for unilateral and bilateral cohorts, respectively. Both visual abnormalities and headaches affect around a third of patients in both cohorts. All pre-operative symptom differences between cohorts are not statistically significant (Table 2).

### Surgical Outcome

Weighted pooled incidence of gross total resection (GTR) for the unilateral and the bilateral cohorts are 94.6% (95% CI, 90.7–97.5%; *I*^2^ = 59.0%; *p* = 0.001) and 90.9% (95% CI, 85.6–95.4%; *I*^2^ = 58.1%; *p* = 0.003), respectively. For OGM recurrence, weighted pooled incidence for the unilateral and the bilateral cohorts are 2.6% (95% CI, 0.4–6.0%; *I*^2^ = 53.1%; *p* = 0.012) and 4.7% (95% CI, 1.4–9.2%; *I*^2^ = 55.3%; *p* = 0.006), respectively. For improvement of vision, weighted pooled incidence for the unilateral and the bilateral cohorts are 55.9% (95% CI, 32.4–78.1%; *I*^2^ = 93.3%; *p* < 0.001) and 70.3% (95% CI, 38.2–94.6%; *I*^2^ = 94.2%; *p* < 0.001), respectively. Differences in GTR incidence, OGM recurrence, and vision improvement were not found to be statistically significant (*p* = 0.210, 0.351, and 0.442, respectively). The weighted pooled mean tumor volume for the unilateral and the bilateral cohorts are 57.4 and 71.8 cm^3^, respectively. However, inconsistent tumor volume data (e.g., standard deviation and range) precluded a statistical comparison between these measurements (Tables 3–5).

**Table 3:** Surgical outcomes/complications (unilateral).

**Surgical outcome**	**Weighted pooled estimate (%)**	**95% CI**	***P***	***I*^**2**^ (%)**	**Bias**	
					**Egger's**	**Begg's**
GTR	94.6	90.7–97.5	0.001	59.0	0.271	0.363
Recurrence	2.6	0.04–6.0	0.012	53.1	0.710	0.427
Vision improvement	55.9	32.4–78.1	<0.001	93.3	0.982	0.615
**Complication**						
Hydrocephalus	1.3	0.1–3.3	0.042	41.5	0.182	0.317
Infection	1.2	0.0–2.9	0.192	23.1	0.335	0.415
Stroke	0.0	0.0–0.4	0.988	0.0	0.016	<0.001
Meningitis	0.0	0.0–0.3	0.997	0.0	0.084	0.003
Epilepsy	1.8	0.2–4.1	0.04	42.6	0.409	0.239
CSF leakage	2.7	0.3–6.7	0.006	51.1	0.336	0.147
New-onset anosmia	7.5	0.4–19.8	<0.001	94.1	0.698	0.124
Hemorrhage	0.9	0.0–2.2	0.889	0.0	0.582	0.785
Death	0.1	0.0–0.9	0.684	0.0	0.022	0.004

**Table 4:** Surgical outcomes/complications (bilateral).

**Surgical outcome**	**Weighted pooled estimate (%)**	**95% CI**	***P***	***I*^**2**^ (%)**	**Bias**	
					**Egger's**	**Begg's**
Gross total resection	90.9	85.6–95.4	0.003	58.1	0.991	0.298
Recurrence	4.7	1.4–9.2	0.006	55.3	0.589	0.915
Vision improvement	70.3	38.2–94.6	<0.001	94.2	0.341	0.562
**Complication**						
Hydrocephalus	0.9	0.0–2.9	0.461	0.0	0.062	0.058
Infection	1.4	0.1–3.4	0.219	20.6	0.598	0.125
Stroke	0.0	0.0–0.7	0.823	0.0	0.002	<0.001
Meningitis	1.2	0.1–2.8	0.495	0.0	0.474	0.030
Epilepsy	1.6	0.3–3.5	0.662	0.0	0.328	0.104
CSF leakage	6.3	2.3–11.6	<0.001	76.1	0.037	0.019
New-onset anosmia	9.4	1.0–23.0	<0.001	90.1	0.090	0.052
Hemorrhage	1.9	0.2–4.8	0.037	42.6	0.301	0.470
Death	3.1	0.9–6.1	0.083	34.9	0.768	0.527

**Table 5:** Surgical outcomes/complications (comparison).

**Surgical outcome**	***P***
Gross total resection	0.210
Recurrence	0.351
Vision improvement	0.442
**Complication**	
Hydrocephalus	0.727
Infection	0.851
Stroke	0.583
Meningitis	0.016
Epilepsy	0.858
CSF leakage	0.220
New-onset anosmia	0.810
Hemorrhage	0.150
Death	0.007

### Complications

For both unilateral and bilateral approaches, the most common complication is new-onset anosmia at 7.5% (95% CI, 0.4–19.8%; *I*^2^ = 94.1%; *p* < 0.001) and 9.4% (95% CI, 1.0–23.0%; *I*^2^ = 90.1%; *p* < 0.001), respectively. Similarly, for both approaches, the rarest reported complication is stroke, with 0.0% (95% CI, 0.0–0.4%; *I*^2^ = 0.0%; *p* = 0.988) and 0.0% (95% CI, 0.0–0.7%; *I*^2^ = 0.0%; *p* = 0.823), respectively.

For the majority of complications, weighted pooled incidence between unilateral and bilateral approaches were similar in magnitude: hydrocephalus (1.3 vs. 0.9%; *p* = 0.727), infection (1.2 vs. 1.4%; *p* = 0.851), stroke (0.0 vs. 0.0%; *p* = 0.583), epilepsy (1.8 vs. 1.6%; *p* = 0.858), and new-onset anosmia (7.5 vs. 9.4%; *p* = 0.810). CSF leakage is notable as the pooled estimate from the bilateral approach is more than twice as large as that of the unilateral approach (6.3 vs. 2.7%), though this difference is not significant (*p* = 0.220). Incidence of hemorrhage following bilateral approach surgery was more than 50% greater than that following unilateral surgery (1.9 vs. 0.9%); however, this is also not significant (*p* = 0.150).

**Figure 2:**
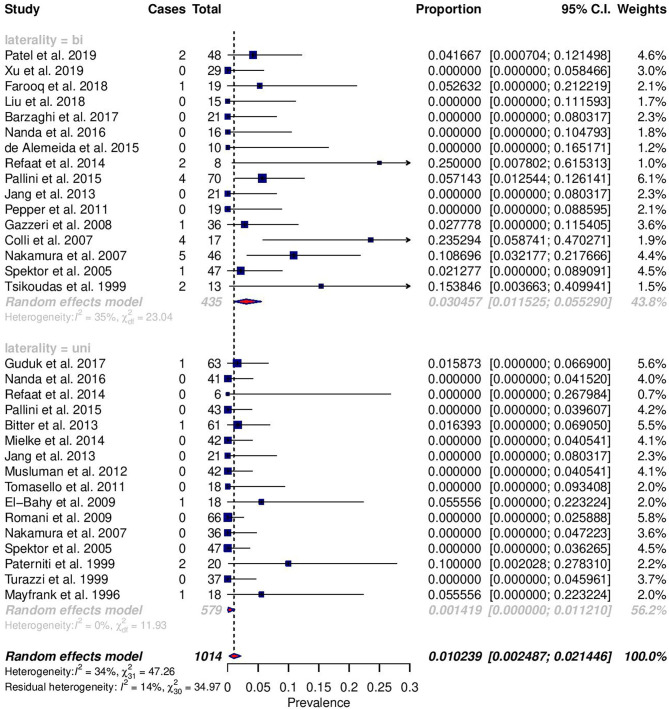
Forest plots comparing the incidence of death between unilateral (uni) and bilateral (bi) approaches.

**Figure 3:**
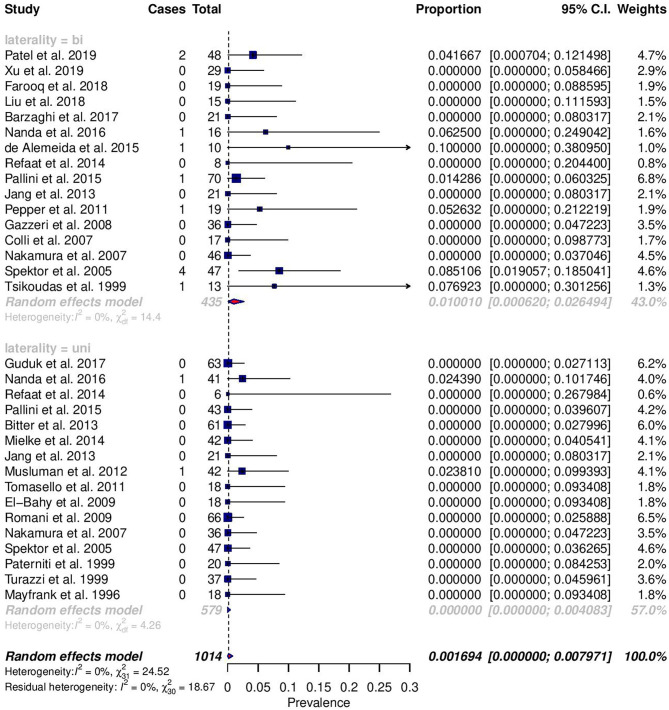
Forest plots comparing the incidence of meningitis between unilateral (uni) and bilateral (bi) approaches.

Among reported complications, only rates of meningitis and death were significantly different between cohorts (Figures 2, 3). For meningitis, the weighted pooled incidence for bilateral approach is significantly greater than that for the unilateral approach (1.2 vs. 0.0%; *p* = 0.016). The bilateral approach's weighted pooled incidence of death is likewise significantly greater than that of the unilateral approach (3.1 vs. 0.1%; *p* = 0.007) (Tables 3–5).

### Meta-Regression

Meta-regression for unilateral and bilateral approach cohorts was performed with covariates of study year, tumor volume, patient age, and study size. Tumor volume and age were significant modifiers (slope = 0.004, *p* = 0.017; slope = 0.02, *p* = 0.007) for the unilateral approach GTR in unilateral surgeries, with volumes and older age associated with a greater proportion of GTR. Both tumor volume and study year were significant modifiers (slope = −0.007, *p* = 0.018; slope = 0.08, *p* = 0.001) for vision improvement in the bilateral cohort, with larger volumes and new studies associated with worse and better vision improvement, respectively. In the bilateral cohort, larger sample size and older age were also linked to greater hydrocephalus incidence (slope = 0.003, *p* = 0.024; slope = 0.02, *p* = 0.008). For both CSF leakage and death, younger age was associated with greater rates of the respective complication (slope = −0.02, *p* = 0.013; slope = −0.02, *p* = 0.014) in the unilateral cohort. For both cohorts, tumor size was negatively correlated with patient age (unilateral/bilateral; slope = −0.99/−0.80), though this relationship was not significant (*p* = 0.410; *p* = 0.756, respectively). The remaining outcomes were unaffected by covariates (Supplementary Material).

### Bias

Given the potential impact of publication bias on meta-analysis findings, funnel plot asymmetry analyses with both Egger's test and Begg's test were performed. Among unilateral approach findings, concern for publication bias was found for stroke and death by both Egger's and Begg's tests and for meningitis by Begg's test alone. With the trim-and-fill method, there are only minor changes to pooled incidence for stroke (0.0–>0.0%), meningitis (0.0–>0.0%), and death (0.2–>0.1%). For bilateral approach findings, concern for publication bias was found for stroke by both Egger's and Begg's tests, CSF leakage by Egger's test alone, and meningitis by Begg's test alone. With the trim-and-fill method again, changes to pooled incidence for stroke (0.0–>0.0%), meningitis (1.0–>0.9%), and CSF leakage (6.3–>6.6%) are minor. Due to model constraints, significance testing was unavailable for the new estimated pooled incidence.

## Discussion

Transcranial resection of OGMs has a long history in neurosurgery. In fact, the first documented success of an intracranial meningioma surgery is an OGM removal with a unilateral approach in 1885 by Durante (1). While a plethora of different and modified approaches have since been developed, a unified consensus with regards to optimal approaches is still lacking. Over time, the strengths and the weakness of popular approaches have become well-characterized.

With broad exposure of the anterior cranial region, bilateral approaches facilitate the removal of hyperostosis from the cribriform area and radical tumor resection. However, it leads to late visualization of critical structures, such as the anterior cerebral/communicating arteries as well as the visual apparatus. In both subfrontal and subcranial approaches, the frontal sinuses often need to be opened, increasing the risk for post-operative CSF leakage. For the subfrontal approach, direct injury to the frontal lobes can occur via retraction for optimal visualization. However, perhaps even more significant, ligation and division of the superior sagittal sinus hinder venous drainage, furthering potential indirect insult to the frontal lobes *via* venous infarction.

**Figure 4:**
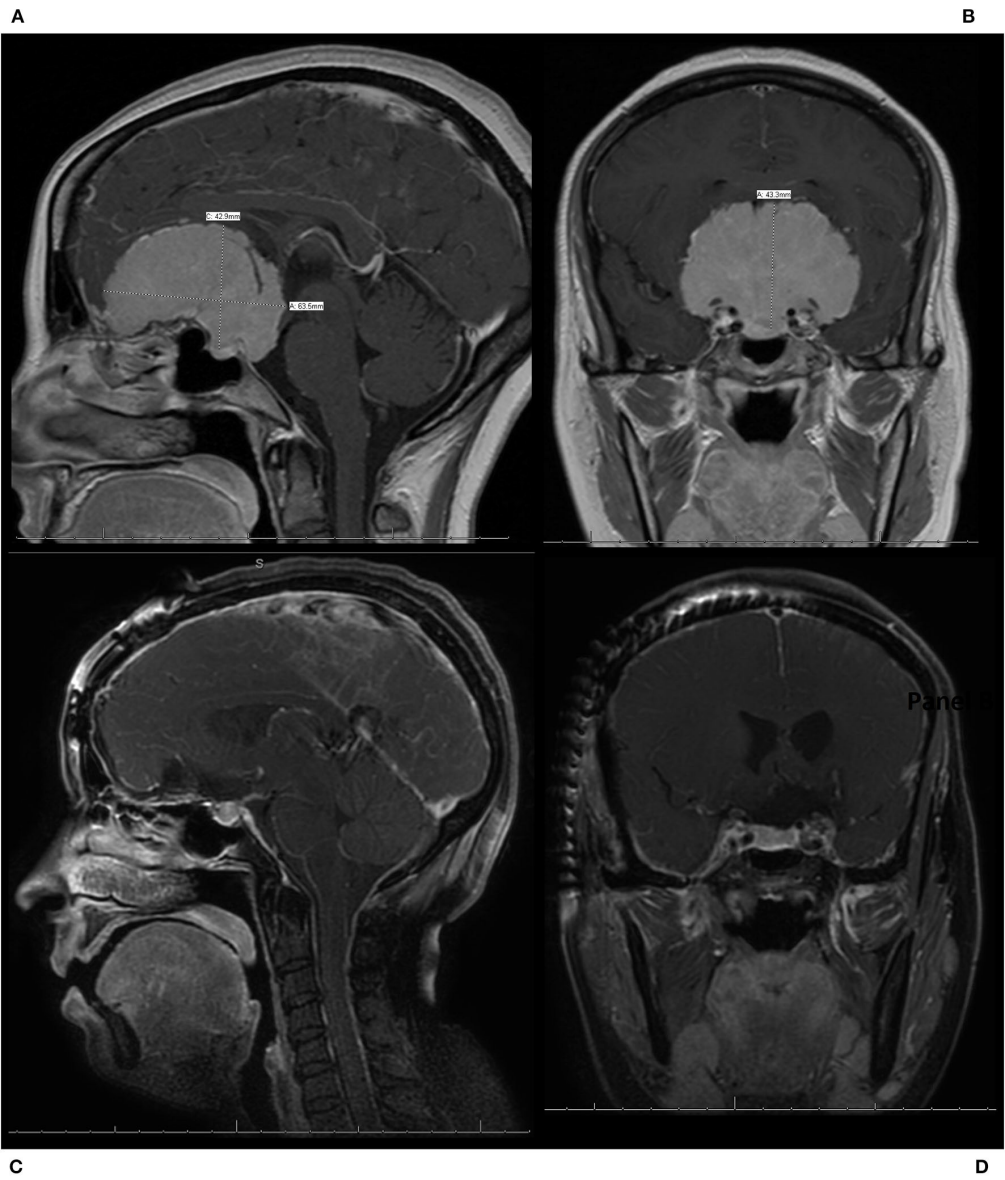
A patient who presented with progressive vision loss and anosmia was found to have a 6.4 cm olfactory groove meningioma (OGM) encasing the bilateral internal carotid artery and its branches as well as the optic nerves bilaterally. The patient underwent a modified pterional craniotomy with extension past midline to expose the superior sagittal sinus for resection of the large WHO grade I OGM. The patient had an immediate improvement in vision post-operatively, with no new neurologic deficits, and was discharged home from the hospital on post-operative day 2. **(A)** Pre-operative sagittal T1 MRI with contrast, demonstrating a large 6.4 cm OGM with encasement of the anterior cerebral arteries and extension in the sella seen. **(B)** Pre-operative coronal T1 MRI with contrast, demonstrating a large OGM with encasement of the internal carotid artery (ICA) and the middle cerebral arteries as well as the optic nerves bilaterally. **(C)** Post-operative sagittal T1 MRI with contrast, demonstrating resection. **(D)** Post-operative coronal T1 MRI with contrast, demonstrating resection with preservation of the ICAs and decompression of the optic nerves.

**Figure 5:**
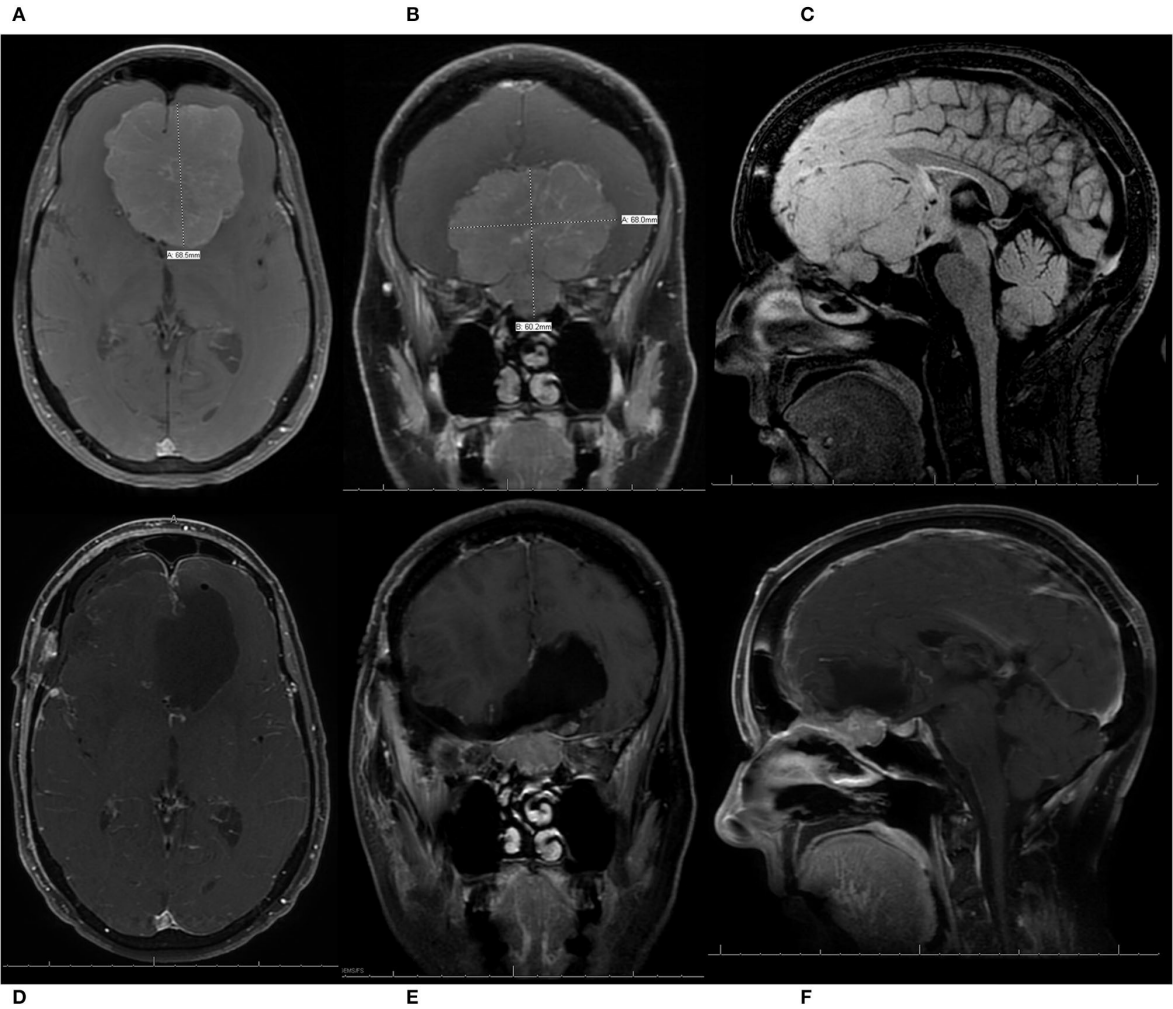
A patient presented with progressive vision loss, anosmia, gait instability, and cognitive decline and was found to have a 6.8 cm olfactory groove meningioma (OGM) with expansion into the endonasal cavity. The internal carotid arteries and their branches were pushed posteriorly by the lesion. The patient underwent a pterional craniotomy for resection of the large WHO grade I OGM with a residual tumor left in the endonasal compartment. The patient had an immediate improvement in vision, with no new neurologic deficits, and was discharged home from the hospital on post-operative day 5. She also enjoyed recovery of taste/smell and gradual but full recovery of her cognition. **(A)** Pre-operative axial T1 MRI with contrast demonstrating a large 6.8-cm OGM with the anterior cerebral arteries (ACAs) pushed posteriorly. **(B)** Pre-operative coronal T1 MRI with contrast, demonstrating a large OGM with extension through the cribriform plate into the endonasal cavity. **(C)** Pre-operative sagittal T1 MRI without contrast, demonstrating a large OGM with endonasal extension and displacement of the ACAs posteriorly. **(D–F)** Post-operative axial, coronal, and sagittal T1 MRIs with contrast, respectively, demonstrating resection of the intracranial component of the large OGM, with preservation of the ACA vasculature and a residual meningioma left in the endonasal compartment to prevent the development of a cerebrospinal fluid leak.

Compared to bilateral approaches, the foremost advantage of unilateral approaches is the ease of approach. Only the ipsilateral frontal lobe is involved, and typically no division of the superior sagittal sinus is necessary. For the pterional approach specifically, the frontal sinuses can be preserved. Visualization and control of the internal carotid artery and optic nerves can also occur earlier. The primary weaknesses of unilateral approaches are reduced access and minimized working angles. The contralateral side of the OGM will always be distant to the surgeon. Excessive manipulation of the frontal lobes may be necessary to properly visualize the tumor (5). The large size and the bilateral extension of many of these lesions would logically presume a wider exposure, and bilateral approach would be the most advantageous. However, it has been the authors' experience that, given the midline origin and the radial growth pattern of these lesions, especially with larger OGMs, the lesions have provided a more-than-adequate exposure and working aperture by pushing the frontal lobe(s) and other critical structures away (Figures 4, 5).

Overall, the findings from this meta-analysis suggest that both approach categories have similar surgical resection outcomes. In terms of tumor resection, the pooled estimated rates of GTR are >90% for both unilateral and bilateral approaches, with no significant differences. In comparison to large, single-institution case series, comparable rates are seen. Nakamura et al. (7) reports GTR rates of 91.2 and 93.5% for unilateral (frontolateral) and bilateral (bifrontal) approaches in 76 patients. For their cohort of 99 patients, Pallini et al. (8) reports 84.8 and 81% of GTR with unilateral (pterional) and bilateral (bifrontal + fronto-orbito-basal) approaches. In terms of recurrence, the pooled estimated rates for bilateral approaches were greater than the rates for unilateral approaches, but the difference was insignificant. It is plausible that there is a tendency to choose bilateral approaches for certain tumors (e.g., with paranasal extension) that may have a propensity for recurrence, but further analysis is warranted. Thus, given the importance of achieving GTR as an outcome metric, both approach categories are similarly effective for OGM removal.

It is common knowledge that tumor volume is a key consideration for approach selection, with bilateral approaches providing more sizable operating fields for larger tumor removal. Though statistical analysis could not be performed, it was noted that the weighted pooled tumor volumes for the bilateral approaches were larger than the unilateral ones. Interestingly, tumor volume was negatively and positively correlated with GTR for bilateral and unilateral approaches, respectively; only the latter was found to be significant. While these trends appear conflicting, they may not be entirely relevant in practice. The estimated slopes for both approaches are both very minor in magnitude, suggesting that even large variations in volume would only correspond to trivial changes in GTR rates. In conjunction with the fact that both pooled GTR rates are very high, the contribution of tumor volume to GTR may be less vital. Indeed significant risk factors for subtotal meningioma resection were found to be symptomatic presentation and bone invasion, but not tumor volume (33). In the authors' experience, size has never been a limiting or deciding factor in the type of approach, and even extremely large tumors can be safely resected via a simple unilateral pterional approach (Figures 4, 5).

The majority of patients of both categories of approaches had visual improvement. Though the difference was not significant, there was a trend of greater improvement in bilateral cohorts. A possible explanation is that bilateral approaches are able to achieve earlier tumor devascularization, facilitating dissection of the tumor away from the optic apparatus. It has been previously reported that the EEA has superior rates of vision improvements compared to TCAs. In particular, Kitano et al. (34) specifically report a significant improvement of visual acuity with EEA, but not for visual field defects compared to TCA. Though the EEA outcomes are outside the scope of this study, it is notable that vision improvement is not reported as a singular outcome. In our systematic review, the heterogeneity of reporting precluded such specificity in defining visual improvement. However, given the importance of vision to quality of life, future investigation on the relationship of specific approaches with post-operative visual function could provide important insights.

In terms of complications, bilateral and unilateral approaches have similarly low rates, of which most were found to be insignificant. This suggests that many of these complications were not consequences of the specific approach but likely inherent to undergoing craniotomy in general. Select complications were still found to be different between categories. Although a significant difference was not found, the bilateral category's pooled estimate of CSF leakage was markedly greater than the unilateral category's rate. As the bilateral opening of frontal sinuses is an inherent step of bilateral approaches, it is not unexpected to observe this trend. Additionally, of other possible contributing factors, orbital osteotomy, either unilateral or bilateral, is known to improve tumor exposure at the risk of increased CSF leakage (1). It may be worthwhile to further examine the utility of this trade-off given the procedure's association with CSF leakage, which is also linked to additional complications like headaches and meningitis.

Only the complication rates of meningitis and death were significantly different, and both were higher in the bilateral category. As such, the higher rates of CSF leakage in bilateral approaches may explain the higher rates of meningitis. Additionally, risk factors for post-craniotomy meningitis include longer duration of drain placement, longer length of surgery, and ICU admission—clinical parameters which are more likely to be associated with the larger involvement of bilateral approaches (35). Greater size and invasiveness of bilateral approaches are likewise likely primary contributors to greater mortality, subsequent to the development of post-operative brain edema (7, 8). Though not often reported, the specific causes of death are elucidating. Pulmonary embolism was seen in both unilateral and bilateral categories, suggesting that it is a non-specific consequence (7, 20, 27). However, given the fact that bilateral approaches are generally larger and involve more procedures, the increased duration of surgery would expose patients to higher risks of thromboembolism (36). Of the deceased patient who received a bilateral approach, Spektor et al. (5) describes CSF rhinorrhea leading to meningitis and death. Two of the deaths, also seen associated with bilateral approaches, reported in Nakamura et al. were caused by hemorrhage and edema (7). Notwithstanding these singular examples, they suggest how bilateral approaches can be riskier.

Given their significance relative to other complications, death and meningitis may be occurring in a subpopulation of OGM patients with different tumor characteristics from the overall population. For instance, these patients could have had larger and more aggressive tumors, necessitating radical cranial base resection—a choice better suited for bilateral approaches but one that increases the risk for CSF leaks. Pallini et al. (8) qualitatively comments on the larger size of these tumors in the patients who died. Another possible difference is age, which was found to negatively correlate with tumor size across the analyzed studies. Lu et al. (6) report a similar trend for patient age and anterior skull base meningiomas (e.g., olfactory groove and tuberculum sellae), and though our trend was not significant, this relationship may manifest more clearly as the literature grows.

## Limitations

Although our study was conducted according to the PRISMA guidelines, there are a few limitations to this meta-analysis. Foremost, there are no RCTs and only minimal comparative studies of TCAs. This deficiency in the literature meant that the only available types of studies for meta-analysis were case series, which are relatively low in the hierarchy of evidence quality. Additionally, without direct comparisons of TCA cohorts, odds ratios have not been calculated, and a meta-analysis of proportions was performed instead. To ameliorate these weaknesses, strict criteria for inclusion and exclusion were implemented and followed to maximize data quality. Furthermore, RE modeling was used for all analyses, given the heterogeneity and the variability in both studies (e.g., publication year, country, and duration) and clinical characteristics (e.g., surgeon experience and skill, post-operative management). While promising that our conclusions closely mirror the largest two OGM case series, this meta-analysis still needs to be interpreted with greater caution, given its source material.

Small sample sizes are another limitation for most of the included studies. Especially for rarer complications such as stroke, a limited cohort size may not be able to capture their true incidence. As a result, artificially low rates may be erroneously reported. There are also often varied levels of clarity in the reporting of outcomes and complications. Though analyzing multiple studies theoretically overcomes this noise and imprecision, it is still a potential error that could be eliminated by standardized assessments and measurements. Another issue is possible inconsistencies with clinical assessments, particularly for nuanced complications like anosmia. Out of 27 studies, only Jang et al. described an objective scale for olfactory evaluation. Discrepancies in assessment could hinder both the accuracy and the statistical significance of our findings. Finally, akin to reporting variability, selection bias for approach is a factor that is difficult to account for. Despite the general principles for choosing an approach, the lack of consensus-driven criteria explains its existence. As most of these concerns stem from working with case series, they can be overcome through higher-quality study types like RCTs or prospective cohort studies, being performed in the future, that utilize objective evaluations of patient complications.

## Conclusion

Multiple TCAs are utilized for surgical resection of OGMs. Though a plethora of approaches exist, they may be simply categorized into unilateral or bilateral approaches. Through a systematic review and meta-analysis of proportions, it was found that, though comparable in many aspects of surgical outcomes and complications, bilateral approaches had a significantly higher risk of post-operative meningitis and death compared to unilateral ones. Though these insights need to be interpreted carefully, they suggest that unilateral approaches may be safer for the resection of OGMs. Given the presence of multiple comparative studies between EEA and TCA, the paucity of studies analyzing specific TCAs is unfortunate. This topic should be explored in greater depth with larger studies.

## Data Availability Statement

The original contributions presented in the study are included in the article/Supplementary Material, further inquiries can be directed to the corresponding author.

## Author Contributions

AF and SW authored the manuscript. AF, SW, and SS performed study selection and appraisal. MJ and AT provided analytical input and assistance with study. AP, AH, PR, and AE supervised the study. All authors agreed to submitted version of manuscript.

## Conflict of Interest

The authors declare that the research was conducted in the absence of any commercial or financial relationships that could be construed as a potential conflict of interest.
